# A taxonomy of cyber risk taxonomies

**DOI:** 10.1111/risa.16629

**Published:** 2024-08-02

**Authors:** Giovanni Rabitti, Amir Khorrami Chokami, Patrick Coyle, Ruben D. Cohen

**Affiliations:** ^1^ Department of Actuarial Mathematics and Statistics Heriot‐Watt University and Maxwell Institute for Mathematical Sciences Edinburgh UK; ^2^ Department of Mathematics and Computer Science Università di Cagliari Cagliari Italy; ^3^ Aviva Ireland Dublin Ireland; ^4^ Operational Risk and Reinsurance Solutions Howden Group Holdings London UK

**Keywords:** cyber risks, industrial taxonomy, risk classification

## Abstract

The field of cyber risks is rapidly expanding, yet significant research remains to be conducted. Numerous taxonomy‐based systems have been proposed in both the academic literature and industrial practice to classify cyber risk threats. However, the fragmentation of various approaches has resulted in a plethora of taxonomies, often incongruent with one another. In this study, we undertake a comprehensive review of these alternative taxonomies and offer a common framework for their classification based on their scope. Furthermore, we introduce desirable properties of a taxonomy, which enable comparisons of different taxonomies with the same scope. Finally, we discuss the managerial implications stemming from the utilization of each taxonomy class to support decision‐making processes.

## 1 INTRODUCTION

Over the past decade, there has been a consistent increase in well‐documented cyber risk events and losses. This is due to the growing reliance on online data storage and the widespread use of cloud computing, which expose individuals and organizations to a higher risk of cyber incidents. Numerous organizations, including businesses, charities, and government agencies, regularly fall victim to various types of cyberattacks, such as phishing, extortion, and Denial of Service (DoS) attacks. In April 2020, the video communication company Zoom was hacked and over 500K Zoom accounts details were found on the dark web either published or for sale.^1^ The Health Service Executive, the health care system in Ireland, also fell to a massive data breach. According to PwC (2021), “*This immediately resulted in healthcare professionals losing access to all HSE provided IT systems ‐ including patient information systems, clinical care systems and laboratory systems. Non‐clinical systems such as financial systems, payroll and procurement systems were also lost*.” The hackers demanded a payment of more than £14 million in exchange for decrypting the data and refraining from publishing it online. In April 2019, the technology company Facebook (now known as Meta) experienced a data breach that led to the theft of information from more than 533 million Facebook users. The stolen information included Facebook IDs, account names, email addresses, comments, and like reactions.^2^ These enormous amounts of illicitly accessed data can be sold on various criminal marketplaces for profit. Stolen data are often used to commit further crimes like encouraging fake bank transfers, breaking into other accounts (phishing), and extortion via DoS.

Despite the ongoing trend, only a limited amount of data concerning losses from cyber risks have become available. As a result, the concept of cyber risk is considered an emerging risk (Dacorogna & Kratz, 2022). Continuous research is being conducted to enhance our comprehension of the insurability (Biener et al., 2015; Malavasi et al., 2022), effects (Eling & Jung, 2022; Welburn & Strong, 2022), expenses (Eling & Wirfs, 2019; Eling et al., 2023), and approaches to mitigating or preventing cyber risks (Paté‐Cornell et al., 2018). As a result, the pricing of cyber insurance coverage is still in its early stages (Xu & Hua, 2019), and the cyber insurance market is rapidly developing (Peters et al., 2018).

A notable challenge in modeling cyber risks is the absence of standardized terminology within the field of cybersecurity (Cains et al., 2022). Aldasoro et al. (2020, p. 376) write that “*An accurate quantification of cyber risks […] is challenging, as there is no precise definition of cyber events*.” Both researchers and practitioners are actively seeking an appropriate taxonomy to define and classify cyber risk (Dacorogna & Kratz, 2023). Organizations require a clear, transparent, universally applicable taxonomy for cyber risks that is easily comprehensible. A robust taxonomy aids analysts and risk managers in effective preparation and implementation of risk controls, mitigation strategies, and procedures that can significantly reduce the impact of cyberattacks. The presence of such a well‐defined taxonomy is crucial because it empowers organizations to make well‐informed decisions about the necessary actions to take, based on the specific category of the cyber risk, the likelihood of its occurrence, and the potential impacts. Conversely, the adoption of an inadequate taxonomy can lead to uncertainty in accurately labeling cyber events and hinder their statistical analysis by introducing heterogeneity. Manual classification of cyber events is susceptible to human misjudgment and uncertainty. This uncertainty poses a significant challenge for insurance companies, as they may struggle to accurately price the risk events, thus exposing themselves to underwriting risk. Failure to address this uncertainty could also lead to modeling risk, where the actuarial models may not accurately reflect the risks being modeled. This evidences the fact that without a proper terminology and taxonomy, quantifying cyber risk becomes even more complicated.

As noted by Dacorogna and Kratz (2023), the selection of a taxonomy for classifying cyber risks has significant implications for the collection and interpretation of cyber loss data. The authors emphasize that while governmental bodies might favor a finely detailed taxonomy for monitoring and implementing precise risk management strategies, insurance firms frequently utilize simpler taxonomies featuring fewer event types. Consequently, having a reduced number of cyber loss categories leads to an increase in the observations within each category. Dacorogna and Kratz (2023) conclude that “*some more work [on the taxonomy] is needed*” (p. 8).

In this study, we propose a classification of taxonomies in order to provide a comprehensive framework for their utilization. We believe this topic is important for the subsequent statistical analysis of cyber risk because, given the multitude of taxonomies dispersed throughout the literature, formalizing their types can greatly benefit cyber risk analysts. Our work aligns with the perspective presented in Cremer et al. (2022), where “*a plea is made for open data and the standardisation of cyber risk data for academic comparability and replication*” (p. 701).

Precisely, we have identified four types of taxonomies, based on their focus and use:

1. Attack‐based taxonomies;
2. Harm‐based taxonomies;
3. Operational risk taxonomies; and
4. Holistic taxonomies.

Moreover, we also discuss a good set of desirable properties that these taxonomies should satisfy, with the aim of simplifying the comparison among taxonomies.

This paper is organized as follows. In Section 2, we present the four types of taxonomies adopted both in the academic literature as well as in the industrial practice. In Section 3, we discuss desirable properties. Section 4 discuss the managerial implications of a choice of a taxonomy.

## 2 CLASSIFICATION OF TAXONOMIES

As previously mentioned, the literature on cyber risks has been relatively modern due to the availability of meaningful data and varying interpretations of events (Romanosky, 2016). However, Cohen et al. (2019) highlight the lack of consistency across taxonomies and models, emphasizing the need to gain a better understanding of this risk. Analogously, Eling et al. (2021) highlight the lack of uniformity in the academic literature's categorization of cyber incidents. These authors emphasize that the identification of cyber risk events remains an ongoing and critical area for future research, as it can influence the success of risk management strategies. Based on considerations of purpose and usage of a taxonomy, we propose the classification described in the following subsections.

### 2.1 Taxonomies based on causes/attack methodology

This class of taxonomies is based on the methods by which a cyberattack is conducted. Adopting a taxonomy on the type of cyberattacks allows organizations to implement tailored risk prevention and mitigation measures. Understanding the methods of attack facilitates the identification and mitigation of potential vulnerabilities in information systems, leading to the development of effective response strategies in the event of an attack. This taxonomy can also assist in training personnel to recognize warning signs and adopt safer online behaviors. This may include training staff to recognize phishing techniques or to avoid clicking on suspicious links. Moreover, investments in the IT sector have been shown to reduce the impact of cyber events (Aldasoro et al., 2022).

In the cybersecurity literature, taxonomies based on the type of attack have been classified in various works (see Juliadotter & Choo, 2015; and Unterkalmsteiner & Adbeen, 2023). However, the highly specific language and technical use of terminology make these approaches too specialized for general cyber risk management within organizations. While a technical language is crucial for effective communication among IT professionals, it may not facilitate decision‐making for nontechnical individuals, such as risk managers.

The work of Romanosky (2016) offers a good example for this type of taxonomies in terms of cyber incident types described in Table 1.

**TABLE 1:** Cyber incidents of Romanosky (2016).

Data breach	Security Incident	Privacy violation	Phishing/ Skimming

Other taxonomies extend in different directions, especially in terms of increasing the causes of cyber incidents.

There are taxonomies of this type stemming from the nonacademic sectors. A first example is offered by the Privacy Rights Clearinghouse (PRC) data set.^3^ It is a public data set of data breaches occurring in the United States, starting from 2005, with the latest observation being in 2019. The PRC data categorize cyber risk by breaches to the organization with the types of breach and descriptions detailed in Table 2.

**TABLE 2:** Table of Privacy Rights Clearinghouse (PRC) Cyber Taxonomy, going by type of breach.

Type of breach	Description
**CARD**	Fraud involving debit and credit cards Not via hacking (skimming devices at point‐of‐service terminals, etc.)
**HACK**	Hacked by an outside party or infected by malware
**INSD**	Insider (employee, contractor, or customer)
**PHYS**	Physical (paper documents that are lost, discarded, or stolen)
**PORT**	Portable device (lost, discarded, or stolen laptop, PDA, smartphone, memory stick, CDs, hard drive, data tape, etc.)
**STAT**	Stationary computer loss (lost, inappropriately accessed, discarded, or stolen computer or server not designed for mobility)
**DISC**	Unintended disclosure not involving hacking, intentional breach or physical loss (sensitive information posted publicly, mishandled or sent to the wrong party via publishing online, sending in an email, sending in a mailing, or sending via fax)
**UNKN**	Unknown (not enough information about breach to know how exactly the information was exposed)

Another example from a nonacademic source is found in the cybercrime report issued by the Federal Bureau of Investigation (FBI), which employs a taxonomy known as the IC3 to categorize these crimes. *The FBI's Internet Crime Complaint Center (IC3) provides the American public with a direct outlet to report cybercrimes to the FBI* (Federal Bureau of Investigation, 2021). The IC3, established in May 2000, has received over 6.5 million complaints to date. It serves as a mechanism to gather intelligence on cyber and Internet crime to help businesses and organizations better understand the variety of threats they are exposed to. Compared to the PRC taxonomy, the FBI taxonomy contains more categories (31 in total), that we report in the Appendix. This taxonomy contains innovative aspects compared to previous ones, as it addresses cyber events that also affect a person's social and professional spheres. For instance, the categories “Employment” and “Romance fraud” involve fraudulent online payments from individuals who believed they were working for a fictitious company or were in a virtual relationship with someone who did not actually exist.

Another example of a taxonomy in this class is the one adapted by PwC (2022), which uses the MITRE ATT&CK framework as a taxonomy for techniques of entry into the system. This was the most relevant and up‐to‐date guide (see Table 3).

**TABLE 3:** PwC categories of their MITRE ATT&CK framework.

Initial access	Execution	Persistence	Privilege escalation	Defense evasion
Discovery	Collection	Command and Control	Resource / Development	

Table 3 contains nine categories of cyber risk taxonomy using technique of cyber event as the principle for categorizing them. This taxonomy breaks down the process of a cyberattack into distinct stages and actions, providing a detailed framework for understanding and analyzing each phase of an attack.

### 2.2 Taxonomies based on potential harm

In this subsection, we explore taxonomies constructed based on the potential damages and/or consequences of a cyberattack. The benefits of this type of taxonomies are evident in its capacity to quantify monetary damages, thereby, for example, facilitating the development of insurance products. For a taxonomy of this type, organizing the losses on an ordinal scale might allow for the sharing of cyber data. However, this could decrease the accuracy of the model's predictions based on these data (Giudici & Raffinetti, 2022).

In general, it is worth emphasizing that the use of these types of taxonomies applies to cyber risk loss after an attack, irrespective of the attack methodology. Consequently, numerous financial and insurance firms have adopted this taxonomy. Furthermore, it is notable that within this taxonomy, an attack can yield diverse damages, potentially triggering a cascade of losses. In this category, we consider as a first example the taxonomy adopted by AXA Insurance accessible in its documentation (AXA, 2009). As shown in Table 4, it is worth noting that this taxonomy offers a framework for categorizing the most prevalent types of cyber impact. Moreover, legal and regulatory compliance can lead to losses explicitly incorporated in this taxonomy.

**TABLE 4:** AXA cyber risk taxonomy, which deviates from the PwC taxonomy.

**Financial**	Most companies are primarily motivated by profit, therefore there is often a large emphasis on financial impact. However, other scenarios, such as those listed below, can have an indirect financial impact and capturing these overlapping impacts can prove challenging. Sometimes loss of revenue due to business interruption outweighs direct financial losses when funds are redirected or stolen.
**Reputational**	Cyber incidents can also erode reputations, thereby damaging the customer's trust in the organization and their willingness to continue supporting it. This can lead to a loss of customers and revenue, which can then result in a reduction in profits.
**Legal/ Regulatory**	Data protection laws are becoming increasingly sophisticated as the world becomes more digitized. If an organization fails to protect the personal information it holds, it may face fines and regulatory actions.

The report of U.K.‐Marsh (2015) includes another taxonomy developed by Marsh in collaboration with the UK government, reported in Table 5. In Table 5, we observe there is no regulatory compliance category, while other categories have been expanded into more granular categories.

**TABLE 5:** Harm taxonomy from U.K.‐Marsh (2015).

Loss category	Description
Intellectual property (IP) theft	Loss of value of an IP asset, expressed in terms of loss of revenue as a result of reduced market share.
Business interruption	Lost profits or extra expenses incurred due to the unavailability of IT systems or data as a result of cyberattacks or other nonmalicious IT failures.
Data and software loss	The cost to reconstitute data or software that has been deleted or corrupted.
Cyber extortion	The cost of expert handling for an extortion incident, combined with the amount of the ransom payment.
Cybercrime/Cyber fraud	The direct financial loss suffered by an organization arising from the use of computers to commit fraud or theft of money, securities, or other property.
Breach of privacy event	The cost to investigate and respond to a privacy breach event, including IT forensics and notifying affected data subjects. Third‐party liability claims arising from the same incident. Fines from regulators and industry associations.
Network failure liabilities	Third‐party liabilities arising from certain security events occurring within the organization's IT network or passing through it in order to attack a third party.
Impact on reputation	Loss of revenues arising from an increase in customer churn or reduced transaction volumes, which can be directly attributed to the publication of a defined security breach event.
Physical asset damage	First‐party loss due to the destruction of physical property resulting from cyberattacks.
Death and bodily injury	Third‐party liability for death and bodily injuries resulting from cyberattacks.
Incident investigation and response costs	Direct costs incurred to investigate and “close” the incident and minimize postincident losses. Applies to all the other categories/events.

It is possible to consider loss taxonomies only for certain specific types of attacks and integrate them accordingly based on the type of attack. For instance, Allianz employs the taxonomy for losses following ransomware attacks as outlined in Table 6.

**TABLE 6:** Allianz Taxonomy for the ransomware attacks in terms of the number of extortions (Allianz, 2022).

Single extortion	Double extortion
Extortion payment: Demanded by criminals.	Notifications costs: Notifying customers, regulators, and other required authorities of a data breach.
Lost income (Business interruption): The longer the period of time in which system accessibility is limited, the greater the loss.	Monitoring costs: Monitoring services for identity theft/fraud that must be supplied to individuals whose data are stolen.
Recovery expenses: The cost of restoring data and ensuring full systems recovery.	Regulatory fines and legal expenses: Due to third parties' claims whose private data are stolen.
Forensics expenses: Expenses incurred to investigate the source of the security vulnerability.	Data recovery and PR repairment: Costs of a consultant, crisis management firm, or law firm to limit the effects of negative publicity.

There are also academic contributions that consider taxonomies of this kind. Agrafiotis et al. (2018) take a distinctive approach to cyber risk analysis by evaluating and quantifying the potential harm that organizations may face. It highlights the current lack of strong incentives to heavily invest in cybersecurity infrastructure and practices, and suggests that businesses should conduct a cost–benefit analysis of the associated risks and harms. Their taxonomy of cyber harm events encompasses five overarching themes in Table 7.

**TABLE 7:** Taxonomy of cyber harms of Agrafiotis et al. (2018).

Physical or digital harm	Economic harm	Psychological harm	Reputational harm	Social harm	Societal harm

The article by Agrafiotis et al. (2018) presents a noteworthy contribution in the form of a taxonomy for cyber harm exposure, which is distinct from other literature reviewed for this study and provides valuable insights. These authors discuss various approaches to identifying cyber risk arising from attacks, acknowledging that a harm‐based taxonomy alone is not comprehensive but serves as a foundational framework that can be further developed by considering different stakeholders' exposures and impacts. These perspectives have implications for the employed cyber harm quantification model.

A taxonomy for potential harm can be used to support quantification of the severity from a legal perspective of cybercrime. For instance, the French National Police Force (Gendarmerie Nationale) condensed 475 types of cybercrimes into the following taxonomy^4^ reported in Table 8. With respect to previous taxonomies in this class, we note the interesting feature that it includes crimes against the Nation and the State, highlighting the fact that the focus of this taxonomy pertains the national security level.

**TABLE 8:** Crimes and offenses according to the French government.

Category	Description
Crimes and offenses	Attacks on the physical or psychological integrity of the individual
against persons	Attacks on the dignity of the individual
	Attacks on personality
	Offenses against minors and the family
	Offenses in the field of the press
Crimes and offenses	Destructions, degradations, and deteriorations
against property	Attacks on automated data‐processing systems (Articles 323‐1 to 323‐7 of the Penal Code)
	Infractions of the Postal and Telecommunications Code – Electronic communications
	Infractions of the Intellectual Property Code
	Fraudulent capture of televised programs (Articles 79‐1 to 79‐6 of Law nG86‐1067 of September 30, 1986)
Crimes and offenses	Attacks on the fundamental interests of the nation
against the nation,	Attacks on the authority of the State
state, and public peace	Attacks on public confidence

Phillips et al. (2022) develop a hierarchical classification of cybercrimes, representing them as a tree structure. This classification, which aligns with the three layers of cyberspace, was accepted by the European Commission in 2013, albeit with minor variations in terminology. The categories are:

- Crimes against machine: This encompasses offenses specific to computers and information systems within the European Union (EU).
- Crimes using the machine: This category refers to traditional offenses that involve the utilization of computers or information systems within the EU.
- Crimes in the machine: It covers content‐related offenses within the EU.
- Incidental technology use
- Organized crime, Deep Web markets, Illegal virtual marketplaces, and Cybercrime‐as‐a‐Service
- Information and behavioral manipulation

A possible drawback of these taxonomies is that the classification of losses might not be clear. For instance, the Open Threat Taxonomy (OTT) in Tarala and Tarala (2015) *only describes threat actions, but uniquely includes a priority ranking for each action*. The losses it considers include 75 types of threats of four categories: Physical, Resource, Personnel, and Technical threats. However, it is worth noting that it does not specifically address cyberattacks; instead, it includes natural risks, and due to its descriptive nature, it may lead to classification ambiguity without precise definitions. This aspect is also discussed in Launius (2021).

### 2.3 Taxonomies based on operational risk

A common approach to model cyber risk is based on its shared features with the operational risk. Operational risk is commonly defined as: “*The risk of losses resulting from inadequate or failed internal processes, people and systems; or from external events*.” On this line, Strupczewski (2021) defines the cyber risk as “*an operational risk associated with performance of activities in the cyberspace, threatening information assets, ICT resources and technological assets, which may cause material damage to tangible and intangible assets of an organisation, business interruption or reputational harm. The term ‘cyber risk’ also includes physical threats to the ICT resources within organisation*.” This comprehensive definition serves as a foundational background for the risk classification framework employed in this subsection. The aim of this type of taxonomy is to categorize all potential causes of operational disruptions within a company due to a cyber threat. From a modeling perspective, cyber risk could significantly overlap with operational risks. This alignment encompasses risks related to people (fraud, mistakes, and inadequate training), systems (system failure, programming errors), external events and cyber risks (Cohen et al., 2019). In other words, the work of Cohen et al. (2019) demonstrates the significant convergence of operational and cyber risks. Earlier studies, including those by Chavez‐Demoulin et al. (2006), Jarrow (2008), and Aldasoro et al. (2020), have contributed to the understanding, classification, quantification, and modeling of operational risks. Table 9 categorizes operational risks in terms of frequency and severity. This exemplifies the connection between taxonomy and risk severity. The same event can be classified according to the taxonomy adopted with a certain combination of frequency and severity. This will lead to a statistical estimate of the severity of this event. However, if another taxonomy is adopted, the class of frequency and severity for the same event may be different.

**TABLE 9:** Classification of operational risk taxonomies in terms of frequency and severity.

		Frequency	
		Low	High
**Severity**	**High**	Clients, products, and business practices; Internal fraud; Execution, Delivery and process management;	
	**Low**	Business disruption and system failures;	External fraud; Employment practices and workplace safety; Damage to physical assets;

However, while research on cyber risk has gained attention in recent years (Dacorogna & Kratz, 2022), it remains in its early stages compared to the extensive literature on operational risks.

There are several works whose taxonomies can be framed through parallelism with operational risk. We have summarized these in Table 10, which contains the classes of cyber risk. We did not report the subclasses to facilitate presentation and comparison (e.g., the taxonomy of ENISA (2016) includes 75 subclasses).

**TABLE 10:** Taxonomies based on operational risk.

NIST (2012)	ENISA (2016)	Cebula et al. (2014)	RiskXchange (2023)	Bouveret (2018)	Aldasoro et al. (2020)	U.K.‐Marsh (2015)	Marsh and McLennan (2018)
Cyber or physical attacks Human errors Failure of resources Environmental disasters, accidents, or failures	Physical Attack Unintentional Damages Disasters Failures / Malfunction Outages Eavesdropping / Interception / Hijacking Nefarious Activity / Abuse Legal	Actions of people Systems and technology failures Failed internal processes External events	Internal network risks Employee‐generated risks Social engineering attacks Cloud‐based attacks Third‐party threats	Data Breach Fraud Business Interruption	Unauthorized Activity Internal Theft System Security Internal External Theft and Fraud System Security External Technology & Infrastructure	Actors Capability Persistency Proximity of the attacker Point of attack Type of damage	Business interruption and Operational disruption Reputational harm Employee exposures Regulatory compliance

As it can be observed from Table 10, there is a common framework for these taxonomies based on the rationale of operational risk. In particular, they include both elements of possible harm to the company as well as attack types (as external events), seen as causes of business interruptions. Hence, this setting for a taxonomy can be seen as an hybrid approach between those presented in Subsections 2.1 and 2.2.

Table 10 highlights various aspects of these taxonomies. First, the same cyber event can be categorized into different classes or subclasses. For example, in the taxonomy in U.K.‐Marsh (2015), the type of attack, the attack target, and the type of loss are categorized separately. Moreover, consider that if a phishing attack occurs and someone clicks on the link, according to the taxonomy of Cebula et al. (2014), this event would be classified as both an “external event” and an “action of people.”

In addition, in Table 10, some authors consider the same database provider (ORX), but with different taxonomies. For instance, the IMF research article by Bouveret (2018) analyzes the cost of cyber risk losses in the financial sector using the ORX database. Precisely, Bouveret (2018, p. 10) regards cyber losses as a function of *(Threat, Vulnerability, Consequences)*. The same ORX database is utilized in Aldasoro et al. (2020), where cyber events are classified in terms of operational risk events.

Moreover, we note that the taxonomy of Bouveret (2018) is essentially similar but less detailed than the taxonomy in U.K.‐Marsh (2015). This framework is employed for predicting cyber risk in financial institutions.

Finally, Marsh and McLennan (https://www.marshmclennan.com/) offer numerous documents focused on cyber risk analysis. It is of particular interest the publication by Marsh and McLennan (2018), in which “Regulatory Compliance” is included as a cyber risk category presenting an intriguing departure from other taxonomies based on the operational risk. We also note that the taxonomy adopted by AXA (based on potential harm, see Table 4) is the only other taxonomy sharing this category.

### 2.4 Taxonomies based on holistic approach

A stand‐alone approach to cybersecurity is represented by the approach of Nai‐Fovino et al. (2019) for constructing a taxonomy for the EU. In the article by Nai‐Fovino et al. (2019), an innovative approach to taxonomy and classification of cyber risks is introduced. The proposed taxonomy adopts a three‐dimensional framework to capture the complexity and multifaceted nature of cybersecurity. The proposed taxonomy supports the definition of cybersecurity terminology across various domains and sectors, capturing all aspects related to the cybersecurity realm. Its aim is to unify and map all the possible entities regarding the multifaceted nature of cybersecurity, integrating expertise across EU competence centers and leading to an increased cybersecurity resilience in the EU. This approach recognizes the need to cluster and organize the diverse aspects of cybersecurity to provide a comprehensive understanding of the field. The specific taxonomies proposed in the paper are defined as follows:

- Cybersecurity domains
- Sectors
- Applications and Technologies

From a practical point of view, we remark that it does not support the quantitative analysis of cyber risk. The proposed taxonomy dimensions do not explicitly provide details about the attack methodology, associated costs, or the extent of harm caused by the risk event. This makes this taxonomy not suitable for the private companies' business (as also observed by the authors). In addition, the overcomplexity of the definitions can make this approach too generic and less useful even from a public point of view of EU centers.

## 3 DESIRABLE PROPERTIES OF A CYBER RISK TAXONOMY

In the past, several authors have worked on analyzing taxonomies for cyber threat events (see, e.g., Juliadotter & Choo, 2015; and Unterkalmsteiner & Adbeen, 2023). In this section, after having presented the settings for the taxonomies for cyber risk, we consider reasonable properties that they should fulfill, regardless of the scope for which the taxonomies have been designed. In particular, we have identified the following set of properties that reasonably characterize a useful taxonomy. Precisely, a taxonomy should be:

1. Complete: any possible cyber event can be characterized.
2. Mutually exclusive: its categories or classifications do not overlap, and each element can be assigned to only one category. In other words, when using a mutually exclusive taxonomy, each entity or item can be placed into only one category without ambiguity or overlap.
3. Clear: easy to understand without additional context.
4. Fragmentable: each class can be divided in two or more subclasses, as the experience of the company increases.

We note that, in practice, a taxonomy may not satisfy all the highlighted properties. Obviously, this does not imply that such a taxonomy is not fit‐for‐purpose, but we believe it might lack versatility over time. For example, a nonfragmentable taxonomy indicates that the availability of new data does not allow for its updating and adaptation. On the other hand, a complete but unclear taxonomy is not useful from a practical point of view, because it creates uncertainty in categorizing cyber events. Furthermore, for a taxonomy used by an insurance company, completeness might not be a generally required property but rather one that is relevant only to the specific cyber events covered by the policy.

We can assess whether some of the taxonomies presented in the preceding section possess these properties. Indeed, this facilitates a comparison of the strengths and weaknesses of different taxonomies in relation to each other.

To begin with, it is worth noting that the taxonomy of Bouveret (2018) presented in Table 10 is not complete since it might not encompass the entirety of the cyber risk landscape, including all its associated risks. Nonetheless, this taxonomy offers a clear distinction of the classes.

On the other hand, Aldasoro et al. (2020, p. 22) write: *Given the nature of the classification, we are not able to accurately capture all the events*, implying that their proposed taxonomy might not be complete. Being solely complete may pose a challenge: the taxonomy outlined in Federal Bureau of Investigation (2020) indeed encompasses all potential cases (completeness); however, it might lack mutual exclusivity, as uncertainty could arise when classifying an attack. We note that in a taxonomy based on operational risk, the same attack can lead to different types of losses, such as physical damage to servers or reputational damage to the company. Mutual exclusivity is therefore a characteristic that may not be satisfied for this entire class of taxonomies. Moreover, a lacking distinction between causes and effects can create uncertainty in classification, as discussed in Launius (2021). For example, the categories in the taxonomy in Tarala and Tarala (2015) are prone to ambiguous interpretation.

We finally remark that there have been proposed taxonomies which are complete but not clear. This is the case of the holistic taxonomy for cybersecurity of Nai‐Fovino et al. (2019, p. 27), who write that their proposed *taxonomy […] might risk to become super‐specialised* due to the *complexity and heterogeneity of the cybersecurity discipline*.

In general, we remark that it is not necessary for the taxonomy adopted by an institution/company to satisfy all of these properties. This might depend on the specific context/aim of its usage. However, considering the discussion above, it facilitates the classification of cyberattacks across institutions.

## 4 DISCUSSION AND MANAGERIAL IMPLICATIONS

A major aim of a good taxonomy is to improve the quality of data collection, statistical analysis, communication of results, and decision‐making. Concerning this last point, one of the key roles that taxonomy plays to help manage and mitigate risk, is to enable the alignment of the underlying risk with insurance. With cyber risk being relatively new and, hence, lesser known and little understood in relation to the more established ones—that is, market, credit, and operational—the design of effective insurance products tends to become increasingly challenging. What adds further to the challenge is that, so far, limited consensus exists between the academic and practical worlds on the taxonomies that are to define cyber risk.

The types and definitions for the taxonomies used in cyber risk abound in the literature, as noted earlier in this paper. They range widely in number, with, for example, the FBI splitting cyber (or Internet) risk into about 35 categories. Since with such a large taxonomy it is difficult to design insurance products, it has, in the interest of practicality, become important nowadays to try to separate the risks and place them into a smaller number of buckets, with the hope that the components across the different buckets remain mutually exclusive and independent.

Furthermore, to help reduce analytical complexity so as to achieve more clarity, it is generally preferred to minimize the number of buckets by tightening the size of the taxonomy. The fine balance between achieving mutual exclusivity across different categories of risk and reducing the number of categories in a taxonomy is indeed critical to gaining a better understanding of cyber risk and enabling its quantification as well as insurability.

The work conducted here analyzes the taxonomy for the specific needs of a company, based on the focus of the decision‐makers. For example, the biggest difference between the taxonomies based on attack and those based on possible harm lies in the direction of the risk management actions: external for building defences against attacks, internal for preventing losses. Namely, once an attack is successful, damages can accumulate in a cascade, hitting multiple sectors of a company. Hybrid taxonomies that aim to capture the full impact of damages in a sequential setting do exist. For example, the taxonomy in U.K.‐Marsh (2015) considers both the characteristics of cyberattacks and their potential damage. This is a feature of some taxonomies mainly based on the operational impact of the cyber risk.

Our research can help not only to extend the scope of the existing academic research, but also to provide a clearer understanding of the levels of impact of the cyber risk so as to enable firms to manage it more effectively. This can help design more focused insurance products, which allow risk quantification in a way that can provide more accurate estimates of premiums and risk pool sizes suited more closely to industry sector and size.

## 5 CONCLUSIONS

In this work, we have conducted a review of the main taxonomies for cyber risk, proposing a classification for them. In addition, we identified some properties that, in our opinion, qualitatively distinguish good taxonomies. We believe that classifying and comparing taxonomies can support the selection of one fit‐for‐purpose taxonomy. A flawed taxonomy can cause the same event to be classified into different frequency and severity classes compared to another taxonomy. Moreover, when the taxonomy is not clear, manual labeling of cyber events is susceptible to human error and uncertainty, especially when the taxonomy is highly granular and categories overlap. Such situations introduce operational risks.

We believe that simplicity should be prioritized over complexity due to the rapid evolution of the sector, which can swiftly render models obsolete in the face of emerging cyber threats against evolving technologies such as blockchain and cryptocurrency.

The development of data‐driven taxonomies using machine learning tools (such as clustering methods) holds promise in the rapidly evolving field of cybersecurity. Leveraging advanced algorithms can reveal patterns and connections within large and intricate data sets, leading to a more comprehensive understanding of cyber threats. However, the effectiveness of such classification methods heavily relies on the quality and quantity of comprehensive cyber data sets that can support these innovative machine learning taxonomies. Investigating taxonomies based on machine learning is an open problem for future research.

## Footnotes

1
https://www.welivesecurity.com/2020/04/16/half‐million‐zoom‐accounts‐sale‐dark‐web/

2
https://techcrunch.com/2019/04/03/facebook‐records‐exposed‐server/

3
https://privacyrights.org/data‐breaches

4
https://www.justice.gouv.fr/lutte‐contre‐cybercriminalite

## CRIME TYPES CATEGORIES FOR THE FEDERAL BUREAU OF INVESTIGATION (FBI)

**Advanced Fee**: An individual pays money to someone in anticipation of receiving something of greater value in return, but instead, receives significantly less than expected or nothing.

**Business Email Compromise (BEC)/Email Account Compromise (EAC)**: BEC is a scam targeting businesses (not individuals) working with foreign suppliers and/or businesses regularly performing wire transfer payments. EAC is a similar scam which targets individuals. These sophisticated scams are carried out by fraudsters compromising email accounts through social engineering or computer intrusion techniques to conduct unauthorized transfer of funds.

**Civil Matter**: Civil litigation generally includes all disputes formally submitted to a court, about any subject in which one party is claimed to have committed a wrong but not a crime. In general, this is the legal process most people think of when the word “lawsuit” is used.

**Computer Intrusion**: Unauthorized access or exceeding authorized access into a protected computer system. A protected computer system is one owned or used by the US Government, a financial institution, or any business. This typically excludes personally owned systems and devices.

**Confidence/Romance Fraud**: An individual believes they are in a relationship (family, friendly, or romantic) and are tricked into sending money, personal and financial information, or items of value to the perpetrator or to launder money or items to assist the perpetrator. This includes the Grandparent's Scheme and any scheme in which the perpetrator preys on the complainant's “heartstrings.”

**Corporate Data Breach**: A data breach within a corporation or business where sensitive, protected, or confidential data are copied, transmitted, viewed, stolen, or used by an individual unauthorized to do so.

**Credit Card Fraud**: Credit card fraud is a wide‐ranging term for theft and fraud committed using a credit card or any similar payment mechanism (ACH, EFT, recurring charge, etc.) as a fraudulent source of funds in a transaction.

**Crimes against Children**: Anything related to the exploitation of children, including child abuse.

**Denial of Service/TDoS**: A Denial of Service (DoS) attack floods a network/system, or a Telephony Denial of Service (TDoS) floods a voice service with multiple requests, slowing down or interrupting service.

**Employment**: An individual believes they are legitimately employed and loses money, or launders money/items during the course of their employment.

**Extortion**: Unlawful extraction of money or property through intimidation or undue exercise of authority. It may include threats of physical harm, criminal prosecution, or public exposure.

**Gambling**: Online gambling, also known as Internet gambling and iGambling, is a general term for gambling using the Internet.

**Government Impersonation**: A government official is impersonated in an attempt to collect money.

**Health Care Related**: A scheme attempting to defraud private or government health care programs which usually involving health care providers, companies, or individuals. Schemes may include offers for fake insurance cards, health insurance marketplace assistance, stolen health information, or various other scams and/or any scheme involving medications, supplements, weight loss products, or diversion/pill mill practices. These scams are often initiated through spam email, Internet advertisements, links in forums/social media, and fraudulent websites.

**IPR/Copyright and Counterfeit**: The illegal theft and use of others' ideas, inventions, and creative expressions—what is called intellectual property—everything from trade secrets and proprietary products and parts to movies, music, and software.

**Identity Theft**: Someone steals and uses personal identifying information, like a name or Social Security number, without permission to commit fraud or other crimes and/or (Account Takeover) a fraudster obtains account information to perpetrate fraud on existing accounts.

**Investment**: Deceptive practice that induces investors to make purchases based on false information. These scams usually offer the victims large returns with minimal risk (Retirement, 401K, Ponzi, Pyramid, etc.).

**Lottery/Sweepstakes/Inheritance**: An individual is contacted about winning a lottery or sweepstakes they never entered, or to collect on an inheritance from an unknown relative.

**Malware/Scareware/Virus**: Software or code intended to damage, disable, or capable of copying itself onto a computer and/or computer systems to have a detrimental effect or destroy data.

**Nonpayment/Nondelivery**: Goods or services are shipped, and payment is never rendered (nonpayment). Payment is sent, and goods or services are never received, or are of lesser quality (nondelivery).

**Overpayment**: An individual is sent a payment/commission and is instructed to keep a portion of the payment and send the remainder to another individual or business.

**Personal Data Breach**: A leak/spill of personal data which is released from a secure location to an untrusted environment. Also, a security incident in which an individual's sensitive, protected, or confidential data are copied, transmitted, viewed, stolen, or used by an unauthorized individual.

**Phishing/Vishing/Smishing/Pharming**: The use of unsolicited email, text messages, and telephone calls purportedly from a legitimate company requesting personal, financial, and/or login credentials.

**Ransomware**: A type of malicious software designed to block access to a computer system until money is paid.

**Reshipping**: Individuals receive packages at their residence and subsequently repackage the merchandise for shipment, usually abroad.

**Real Estate/Rental**: Loss of funds from a real estate investment or fraud involving rental or timeshare property.

**Spoofing**: Contact information (phone number, email, and website) is deliberately falsified to mislead and appear to be from a legitimate source. For example, spoofed phone numbers making mass robo‐calls; spoofed emails sending mass spam; forged websites used to mislead and gather personal information. Often used in connection with other crime types.

**Social Media**: A complaint alleging the use of social networking or social media (Facebook, Twitter, Instagram, chat rooms, etc.) as a vector for fraud. Social Media does not include dating sites.

**Tech Support**: Subject posing as technical or customer support/service.

**Terrorism/Threats of Violence**: Terrorism is violent acts intended to create fear that are perpetrated for a religious, political, or ideological goal and deliberately target or disregard the safety of noncombatants. Threats of Violence refers to an expression of an intention to inflict pain, injury, or punishment, which does not refer to the requirement of payment.

**Virtual Currency**: A complaint mentioning a form of virtual cryptocurrency, such as Bitcoin, Litecoin, or Potcoin.
